# Diagnostic utility of plasma heparin-binding protein and procalcitonin among patients with bacterial infections: a single-center retrospective study

**DOI:** 10.1186/s12879-026-14089-6

**Published:** 2026-08-07

**Authors:** Tao Wang, Yu Wei, Juan Chen, Hao Zhang, Jinjin Chen, Qiang Zhou

**Affiliations:** Department of Clinical Laboratory, The Second Affiliated Hospital of Anhui Medical University, Hefei, Anhui 230601 China

**Keywords:** Heparin-binding protein, Procalcitonin, Immune inflammatory index, Bacterial infection, Diagnostic discrimination

## Abstract

**Objective:**

This study aimed to evaluate the discriminatory capacity of plasma heparin-binding protein (HBP), procalcitonin (PCT), and composite immune inflammatory indices in differentiating bacterial infections from non-infection states, as well as to explore their potential for Gram classification and severity stratification.

**Methods:**

A single-center retrospective case-control study enrolled 65 patients with confirmed bacterial infection and 135 non-infected controls. Plasma HBP, PCT, and complete blood count-derived indices were measured from initial blood samples collected within 24 hours of admission. Group comparisons were performed using statistical tests, and diagnostic performance of each biomarker was evaluated using receiver operating characteristic curves.

**Results:**

HBP levels were significantly higher in infected patients than in controls (*p* < 0.001) and demonstrated the best discriminative performance among all markers (AUC = 0.86). Multivariable analysis identified HBP, monocyte, and lymphocyte count as independent predictors for Gram-positive infections, while HBP, PCT, and neutrophil count were associated with Gram-negative infections. Notably, HBP levels increased progressively from the non-infected group (19.45 ng/ml) to PCT-defined infection localized infection (35.13 ng/ml) and PCT-defined infection severe infection (111.95 ng/ml, *p* < 0.001), supporting its utility in PCT-defined severity stratification.

**Conclusion:**

HBP is a promising biomarker for diagnosing bacterial infections, particularly Gram-negative infection, and for stratifying PCT-defined infection severity. However, given the exploratory nature of the Gram classification findings and the single-center retrospective design, these results require validation in larger prospective cohorts.

**Supplementary Information:**

The online version contains supplementary material available at https://doi.org/10.1186/s12879-026-14089-6.

## Background

Bacterial infections remain a significant clinical challenge worldwide, with incidence and mortality rates consistently ranking among the highest across infectious diseases [1, 2]. If not effectively controlled in a timely manner, localized infections are highly susceptible to rapid progression to sepsis or septic shock, leading to multiple organ dysfunction syndrome and directly threatening patient survival [3]. Therefore, early detection of warning signals indicative of bacterial invasion and objective, accurate assessment of infection severity is crucial for optimizing window for anti-infective therapy and improving patient prognosis [4, 5]. However, current routine diagnostic methods encounter practical bottlenecks: while the isolation and culture of pathogenic microorganisms are recognized as the gold standard for definitive diagnosis, the turnaround time (24 to 72 hours) and suboptimal positive detection rate fail to meet the urgent demand for early and rapid diagnosis [6]. Conventional inflammatory markers, such as white blood cell count and C-reactive protein (CRP), exhibit insufficient sensitivity and specificity in differentiating infectious from non-infectious conditions, and their levels are easily influenced by non-infectious confounders such as trauma and surgery [7–9]. These limitations hinder refined, severity-based stratification in the era of precision medicine. In this context, identifying more efficient, sensitive, and specific biomarkers have become a critical priority in bacterial infection diagnostics [10].

In recent years, several novel biological markers have emerged for the diagnosis of bacterial infections. Heparin-binding protein (HBP), released from the azurophilic granules of activated neutrophils, exhibits rapid mobilization kinetics upon pathogenic invasion, making it a promising early alarm signal for infection [11, 12]. HBP binds to endothelial glycosaminoglycans, activating protein kinase C and Rho kinase pathways, which induce cytoskeletal reorganization and endothelial hyperpermeability, thereby contributing to sepsis-associated organ dysfunction [13, 14]. Existing studies confirm that HBP effectively distinguishes bacterial from viral or non-bacterial inflammatory responses, and its dynamic decline correlates with reduced in-hospital mortality, supporting its diagnostic and prognostic value [15, 16]. Procalcitonin (PCT), a 116 amino acids precursor of calcitonin, is normally secreted at low levels (basal < 0.05 ng/ml) by thyroid C-cells but is markedly upregulated in parenchymal tissues upon bacterial endotoxin and pro-inflammatory cytokine stimulation [17, 18]. PCT levels correlate with infection severity and septic risk, and typically remain near normal during viral infections, offering an advantage in differentiating bacterial from non-bacterial inflammation [19]. Extensive evidence demonstrates that PCT not only discriminates bacterial infections from non-infectious systemic inflammatory but also guides the initiation, de-escalation, and discontinuation of antibiotic therapy, establishing it as an important tool in antimicrobial stewardship [20].

On another front, composite inflammatory indices derived from routine complete blood counts have garnered increasing attention. Four such indices—pan-immune-inflammation value (PIV), the systemic inflammation response index (SII), the novel systemic immune-inflammatory complex index (SIICI), and Systemic inflammation response index (SIRI)—integratively incorporate neutrophil, monocyte, lymphocyte, and platelet counts to assess the dynamic equilibrium between the host’s pro-inflammatory status and immunomodulatory mechanisms [21]. Unlike conventional single-cell parameters, these indices collectively capture the core pathophysiological features of sepsis, characterized by concurrent myeloid overactivation and lymphocyte exhaustion/immunoparalysis. Notably, SIICI positions pro-inflammatory myeloid cells (monocytes and neutrophils) in the numerator and immunoregulatory lymphocytes along with immunothrombotic platelets in the denominator, thereby reflecting the dual inflammatory and immune-suppressive state more comprehensively [21, 22]. These composite indices have demonstrated unique advantages in evaluating the severity of infectious disease [23].

Despite the considerable clinical value demonstrated by each of the aforementioned indicators within their respective application scenario, there remains a notable deficiency in systematic cross-sectional comparisons [24]. The comparative evaluation of the relative merits of various marker combinations is a notable gap in the existing literature, leaving the chain of relevant evidence incomplete [25]. To address these gaps, this single-center retrospective case-control study systematically evaluated and compared the diagnostic utility of plasma HBP, PCT, and four composite inflammatory indices (PIV, SII, SIRI, and SIICI) in patients with bacterial infections, specifically examining whether their combined application yields synergistic diagnostic benefits. We aimed to assess the ability of these biomarkers to differentiate bacterial infections from non-infection states, explore their differential performance in distinguishing between Gram-positive and Gram-negative infections, and evaluate their utility in stratifying PCT-defined infection severity (localized vs. severe) based on PCT thresholds. By integrating novel biomarkers with routine hematological parameters, this study seeks to provide preliminary evidence for optimized biomarker selection and combination in the early diagnosis and severity assessment of bacterial infections.

## Methods

### Study design and data collection

The single-center, retrospective case-control study was conducted at the Second Affiliated Hospital of Anhui Medical University (AMUSH) between August 2025 and February 2026. Clinical data for all enrolled participants were obtained from hospital electronic medical record system, including age, sex, history of underlying diseases, principal admission diagnosis, infection site, clinical outcomes, and the results of pathogenic microbial culture identification and Gram staining. This study was approved by the Ethics Committee of the Second Affiliated Hospital of Anhui Medical University (Ethics Approval No. YSB2025-249). Written informed consent was obtained from all participants or their legal guardians prior to enrollment.

All patients in the infection group had bloodstream infections confirmed by positive blood cultures. These patients were identified through the hospital’s microbiology database rather than ICD‑10 diagnostic codes, with microbiological confirmation being a prerequisite for inclusion. Contaminants (e.g., *Corynebacterium* spp., *Bacillus* spp., coagulase‑negative *staphylococci*) were strictly excluded unless isolated from two or more separate blood culture sets obtained on different occasions, in accordance with standard clinical practice. All patients in the infection group underwent a standardized “two‑bottle bilateral” blood culture protocol, ensuring consistency in microbiological work‑up across all participants. A flow diagram detailing the screening, enrollment, and exclusion processes for both the infection and non-infected groups is provided in Supplementary Figure 1.

Inclusion criteria: Patients were eligible for the infection group if they were aged 18 years or older, hospitalized during the study period, and to have at least one positive blood culture indicating the presence of a recognized bacterial pathogen, along with complete clinical and laboratory data. Contaminants were only accepted if isolated from two or more separate blood culture sets. For the non-infected control group, individuals were eligible if they were aged 18 years or older, hospitalized during the same timeframe, matched to the infection group by sex and age, exhibited no evidence of infection, had no recent surgery or trauma within the past three months, and possessed complete data.

Exclusion criteria: The exclusion criteria for the infection group included confirmed viral or fungal infections, concurrent malignant tumors, hematological disorders, autoimmune diseases, and use of immunosuppressive agents or glucocorticoids within the preceding three months. Pregnant or lactating individuals and those lacking complete medical records were excluded.

Non-infected group: The non-infected group was selected from hospitalized during the same period and matched to the infected group by sex and age. Individuals with inflammatory diseases, significant cardiac or renal dysfunction, or malignancies were excluded, along with those possessing any underlying conditions known to independently affect plasma HBP, PCT, or peripheral blood cell indices. These stringent criteria were implemented to minimize potential confounding factors and spectrum bias.

All laboratory parameters were derived exclusively from the initial venous blood samples collected within the first 24 hours following hospital admission, prior to antibiotics whenever possible feasible. The specific parameters measured included white blood cell, neutrophil, lymphocyte, monocyte, platelet count, HBP, and PCT. All assays were conducted in the hospital clinical laboratory in accordance with standardized operating procedures. Complete blood counts were determined using a XN-10 automatic hematology analyzer, along with its accompanying reagents (Sysmex, Japan), which employs multidimensional analysis technology that combines laser scatter and nucleic acid fluorescence staining. Plasma HBP level were measured using an enzyme-linked immunosorbent assay performed on an AU5800 biochemistry analyzer and its reagents (Beckman, USA). PCT levels were quantified through a direct chemiluminescence method utilizing an Atellica analyzer and its accompanying reagents (Siemens, Germany). For definitive bacterial identification, all positive cultures were processed using matrix‑assisted laser desorption/ionization time‑of‑flight mass spectrometry (MALDI‑TOF MS), The Smart MS 5080 mass spectrometer (Dier Biotech, China) was used for analysis. In summary, clinical specimens, including body fluids or blood were inoculated onto solid agar plates or into liquid blood culture bottles. Solid agar plates were incubated at incubator for 24–48 hours until visible colonies emerged. Blood culture bottles were continuously monitored using an automated blood culture system; upon receiving a positive alarm, the broth was subculture onto solid agar plates and incubated until colony growth was observed. Isolated colonies were then placed on a MALDI‑TOF MS target plate and overlaid with an appropriate matrix solution. When precise laser pulses were applied, the matrix facilitated the desorption and ionization of bacterial proteins. The ionized proteins were accelerated in an electric field, and their time‑of‑flight (TOF) is recorded by a high‑precision detector. Since TOF depends on the mass‑to‑charge ratio of the proteins, each bacterial species generates a characteristic protein mass spectrum. The acquired spectra were compared against a reference database for species‑level identification and the classification of Gram‑positive and Gram‑negative bacteria. MALDI‑TOF MS is a routine, rapid, and reliable method for bacterial identification in clinical microbiology laboratories.

All procedures were conducted in strict adherence to the manufacturers’ instructions. The assay kit exhibited a detection range for HBP of 5.9–300 ng/ml, and In this study, PCT level of ≥0.5 ng/mL was defined as indicating PCT‑defined severe infection, whereas PCT < 0.5 ng/mL were classified as PCT‑defined severe infection. This threshold has been widely validated in the literature as a reliable indicator for assessing infection severity progression and guiding antibiotic therapy [26, 27], and has been adopted as a diagnostic cutoff for severe bacterial infection and sepsis [28]. Based on peripheral blood cell counts, four composite inflammatory indices were calculated using standardized formulas documented in the existing literature [29]: Pan-immune-inflammation value (PIV) = (neutrophil count × platelet count × monocyte count) / lymphocyte count; Systemic immune-inflammation index (SII) = (neutrophil count × platelet count) / lymphocyte count; Systemic immune-inflammatory complex index (SIICI) = (neutrophil count monocyte count × 1000) / (platelet count × lymphocyte count); and Systemic inflammation response index (SIRI) = (neutrophil count × monocyte count) / lymphocyte count. In these formulas, all blood cell counts are expressed in units of × 10^9^ /L, and the derived composite inflammatory indices are dimensionless, presented as numerical values.

### Statistical analysis

All statistical analyses were conducted using R software (v 4.5.1). Continuous variables were assessed for normality using the Shapiro–Wilk test. Normally distributed continuous variables are reported as mean ± standard deviation, with comparisons between two groups evaluated using an independent samples t-test and comparisons among three groups assessed using one-way analysis of variance (ANOVA), followed by post hoc pairwise comparisons using Tukey’s Honest Significant Difference (HSD) test. Non-normally distributed continuous variables are reported as median and interquartile range (IQR: *P*₂₅, *P*₇₅). Comparisons between two groups were performed using the Mann–Whitney U test, while comparisons among three groups utilized the Kruskal–Wallis test, with post hoc pairwise comparisons conducted using Dunn’s test and Bonferroni correction. Categorical variables are presented as frequencies and percentages, with the chi-squared test or Fisher’s exact test applied for group comparisons. All statistical tests were two-tailed, and *p* < 0.05 was considered statistically significant.

To evaluate the discriminatory ability of each candidate marker in distinguishing infection from non-infection, univariate Receiver Operating Characteristic (ROC) analyses were conducted for all continuous variables, including age, neutrophil count, lymphocyte count, monocyte count, platelet count, PCT, HBP, PIV, SII, SIICI, and SIRI. The area under the curve (AUC) with 95% confidence intervals (CIs) was calculated for each marker, and pairwise comparisons of AUCs were performed using DeLong’s test. At the optimal cut-off value determined by the Youden index, sensitivity, specificity, positive and negative predictive values, and likelihood ratios were calculated for HBP.

Given the limited sample size and to mitigate the risk of overfitting, we adopted a conservative modeling strategy. The final diagnostic model for infection discrimination was restricted to a univariate logistic regression incorporating HBP alone, based on its superior performance in univariate ROC analyses. For internal validation, we employed bootstrap resampling with 200 iterations and optimism correction. The optimism-corrected AUC was reported, and model calibration was assessed using calibration curves with 200 bootstrap resamples, accompanied by the Hosmer-Lemeshow goodness-of-fit test.

As an exploratory analysis, a multinomial logistic regression model was fitted with infection type as the three-category outcome (reference: non-infection) to compare Gram‑positive versus Gram‑negative bacterial infections. Collinearity was assessed using the Variance Inflation Factor (VIF); variables with VIF > 10 (PIV, SIICI, SIRI) were excluded, while those with a VIF < 5 were retained. Age and sex were included as covariates, and the likelihood ratio test was used to compare nested models. Model performance was assessed using 10-fold cross-validation, with the mean AUC and standard deviation reported.

For comparisons among the three groups (localized, severe infection, and non-infection), normality and homogeneity of variance were tested; ANOVA/Tukey HSD, Welch ANOVA/Games-Howell, or Kruskal-Wallis/Dunn (with Bonferroni correction) were employed as appropriate. Sex was compared using the by chi-square test. In the infected patient, univariate logistic regression (*p* < 0.10) was used to identify predictors associated with PCT-defined severe infection, with odds ratios (ORs) and 95%CIs reported for each candidate variable. An ROC curve for HBP was plotted to predict severe infection, yielding AUC and optimal sensitivity/specificity based on the Youden index. There were no missing data for any of the variables analyzed.

## Results

### Comparison of indicators between the bacterial infection and non-infected group

Following the rigorous application of the aforementioned inclusion and exclusion criteria, a total of 65 patients were ultimately enrolled in the infection group and 135 patients in non-infected group, resulting in a combined cohort of 200 participants. The two groups were well balanced with respect to baseline characteristics: the mean age of the infection group was 59.22 years, compared to 57.44 years in the non-infected group, with no statistically significant difference observed (*p* = 0.43). In terms of sex distribution, the infection group consisted of 39 males and 26 females, while the non-infected group included 77 males and 58 females, again demonstrating no significant difference between groups (*p* = 0.75), thereby balanced sex composition across both the infection and non-infected groups. Among the 65 patients in the infection group, the most common infection sites were the lung (23, 35.38%), urinary tract (19, 29.23%), and abdomen (15, 23.07%). In terms of culture results, Gram‑negative bacteria were predominant, accounting for 51 isolates (78.46%), with *Klebsiella pneumoniae* (19, 29.23%) and *Escherichia coli* (16, 24.61%) being the most frequently identified pathogens. Gram‑positive organisms were isolated from 14 cases (21.54%), with *Staphylococcus aureus* (12, 18.46%) bing the predominant isolate.

In the comparison of laboratory parameters, HBP level in the infection group was 53.27 ng/ml, significantly higher than the non-infection’s value of 19.45 ng/ml, with this difference being the most pronounced among all indicators examined (*p* < 0.001). The PCT level in the infection group was 0.33 ng/ml, which significantly exceeded the non-infected group level of 0.16 ng/ml (*p* < 0.05). The monocyte count in the infection group was 0.61 × 10^9^ /L, significantly higher than the non-infection‘s value of 0.45 × 10^9^ /L (*p* < 0.05). The neutrophil count in the infection group was 8.04 × 10^9^ /L, significantly elevated compared to the non-infected group’s value of 5.58 × 10^9^ /L (*p* < 0.001). All four composite immune inflammatory indices were significantly elevated in the infection group (all *p* < 0.05): PIV was 693.65 in the infection group versus 508.52 in the non-infected group; SII was 1576.12 versus 959.48; SIICI was 18.65 versus 11.65 and SIRI was 4.51 versus 2.34. No statistically significant differences were observed in platelet count (*p* = 0.68) or lymphocyte count (*p* = 0.82) between the two groups (Table 1).

**Table 1:** Comparison of clinical characteristic parameters between patients with bacterial infection and the non-infection group

Characteristics		Infection （n = 65）	Non-infection （n = 135）	*P*
Age (year,*\bar x**±s*)		59.22 ± 14.49	57.44 ± 16.06	0.43
Gender (*n*)	Female	26	58	0.75
	Male	39	77	
HBP (ng/ml, median, IQR)		53.27 (31.34, 88.46)	19.45 (14.32, 27.82)	<0.001
PCT (ng/ml)		0.33 (0.13, 1.42)	0.16 (0.08, 0.50)	0.01
Monocyte (×10^9^ /L)		0.61 (0.35, 0.84)	0.45 (0.32, 0.64)	0.01
Neutrophil (×10^9^ /L)		8.04 (5.78, 11.72)	5.58 (3.68, 8.02)	<0.001
Platelet (×10^9^ /L)		210.00 (162.00, 286.00)	207.00 (135.00, 265.00)	0.68
Lymphocyte (×10^9^ /L)		1.06 (0.75, 1.60)	1.17 (0.80, 1.60)	0.82
PIV		693.65 (406.58, 2684.25)	508.52 (180.76, 1045.54)	0.03
SII		1576.12 (819.60, 2920.18)	959.48 (513.43, 2161.41)	0.02
SIICI		18.65 (8.20, 41.68)	11.65 (4.78, 24.69)	0.04
SIRI		4.51 (2.12, 9.13)	2.34 (1.02, 5.73)	0.02

PIV:Pan-immune-inflammation value; SII: Systemic immune-inflammation index; SIICI: Systemic immune-inflammatory complex index; SIRI: Systemic inflammation response index

### Differential analysis of indicators across Gram infections

Based on the results of Gram staining, 65 bacterial infections were categorized into two groups: 15 patients with Gram-positive infections and 50 patients with Gram-negative infections. A non-infection (control) group consisting of 135 patients was also included in the study. The Kruskal–Wallis test revealed a significant difference in HBP levels among the three groups (*p* < 0.05) (Table 2). The median HBP level in the Gram-negative group was 63.03 ng/ml, compared to 38.95 ng/ml in the Gram-positive group (Supplementary Table 1). Post hoc pairwise comparisons indicated that only the difference between the Gram-negative group and the non-infected group reached statistical significance (adjusted *p* < 0.05) (Table 2).

**Table 2:** Pairwise comparisons of key indicators among non-infection, Gram-positive, and Gram-negative groups

Indicator	Kruskal-Wallis (*P*)	Dunn’s test with Bonferroni correction (Adjusted *P*)				
	Non-infection VS. Gram-positive VS. Gram-negative	Non-infection VS. Gram-positive		Non-infection VS. Gram-negative		Gram-positive VS. Gram-negative
Age	0.46	0.92		0.52		0.59
Gender	0.48					
HBP	0.01	0.11		0.04		0.77
PCT	0.003	1		0.01		0.78
Monocyte	0.01	0.01		0.26		0.42
Neutrophil	<0.001	0.20		0.01		1
Platelet	0.88	1		1		1
Lymphocyte	0.69	1		1		1
PIV	0.03	0.33		0.05		1
SII	0.02	0.98		0.02		1
SIICI	0.05	0.46		0.09		1
SIRI	0.02	0.30		0.03		1

PIV:Pan-immune-inflammation value; SII: Systemic immune-inflammation index; SIICI: Systemic immune-inflammatory complex index; SIRI: Systemic inflammation response index

Significant differences were observed in PCT levels across the three groups (*p* < 0.05), with values recorded as 0.19 ng/ml in the Gram-negative group and 0.46 ng/ml in the Gram-positive group (Supplementary Table 1). Monocyte and neutrophil counts also exhibited significant differences among the three groups (*p* < 0.05). Among the four composite immune inflammatory indices, PIV, SII, and SIRI demonstrated statistically significant differences across the three groups (*p* < 0.05), while SIICI exhibited a borderline significance level (*p* = 0.05). No significant difference in platelet, lymphocyte counts, or age was detected among the three groups (Table 2).

### The value of HBP in stratifying PCT-defined infection severity

Based on PCT levels, 65 infected patients were categorized into PCT-defined localized infection (*n* = 36, PCT < 0.5 ng/ml) and PCT-defined severe infection (*n* = 29, PCT ≥ 0.5 ng/ml) groups, alongside a non-infection group (*n* = 135 patients). The Kruskal–Wallis test revealed statistically significant difference in HBP levels across the three groups (*p* < 0.001). Bonferroni correction pairwise comparisons indicated that HBP levels differed significantly among all groups (*p* < 0.05), demonstrating a stepwise increase from controls to localized to severe infection.

Among the infected patients, HBP level was significantly higher in the severe group (111.95 ng/ml) compared to the localized group (35.13 ng/ml, *p* < 0.001) (Supplementary Table 2). Additionally, lymphocyte counts were lower in the severe group (0.75 × 10^9^ /L vs. 1.34 × 10^9^ /L, *p* < 0.001). SII was also elevated in the severe group (2010.49 vs. 968.04, *p* < 0.05). No significant differences were observed between the two groups regarding in platelet, monocyte count, PIV, SIICI, SIRI, or age (Supplementary Table 2).

### Construction and validation of models for diagnosing and classifying bacterial infection

To mitigate overfitting risk given the limited sample size (*n* = 200, including 65 infected patients), we conducted univariate ROC analyses for each candidate marker. HBP exhibited the highest AUC of 0.86 (95% CI: 0.80–0.92), followed by neutrophil count (AUC = 0.66, 95% CI: 0.58–0.74) and PCT (AUC = 0.64, 95% CI: 0.56–0.72). All other markers (SIRI, SII, PIV, monocyte count, SIICI, age, platelet count, and lymphocyte count) produced AUCs below 0.64. DeLong’s test confirmed that HBP was significantly superior to all other markers (ΔAUC range: 0.20–0.35, all *p* < 0.001), with the largest difference observed against lymphocyte count (ΔAUC = 0.35, Z = 6.98, *p* < 0.001) and the smallest against neutrophil count (ΔAUC = 0.20, Z = 4.30, *p* < 0.001). At the optimal cut-off value of 40.50 determined by the Youden index, HBP attained a sensitivity of 0.68 (95% CI: 0.56–0.79), a specificity of 0.96 (95% CI: 0.92–0.99), a positive predictive value of 0.88 (95% CI: 0.78–0.96), and a negative predictive value of 0.86 (95% CI: 0.80–0.91). The positive likelihood ratio was 15.23 (95% CI: 8.08–48.73), while the negative likelihood ratio was 0.34 (95% CI: 0.22–0.46). These findings establish HBP as the optimal single biomarker for infection discrimination, outperforming both conventional markers (PCT and neutrophil count) and novel composite indices (SII, SIRI, and PIV).

Given the limited number of infected cases, we simplified our modeling strategy to minimize overfitting. Consequently, we restricted the final model to a univariate logistic regression that incorporates only HBP. Internal validation using bootstrap optimism correction (200 iterations) yielded an optimism-corrected AUC of 0.85 (95% CI: 0.78–0.91), with a minimal drop of 0.01 from the apparent AUC (0.86), indicating low risk of overfitting. Calibration metrics (intercept: 0.12, 95% CI: −0.15 to 0.39; slope: 0.92, 95% CI: 0.75–1.09) and the bootstrap-corrected calibration curve all indicated good agreement between predicted and observed probabilities. The Hosmer-Lemeshow test a yielded non-significant result (*p* = 0.67), which is consistent with adequate calibration; however, its low power in small samples precludes definitive interpretation.

**Fig. 1:**
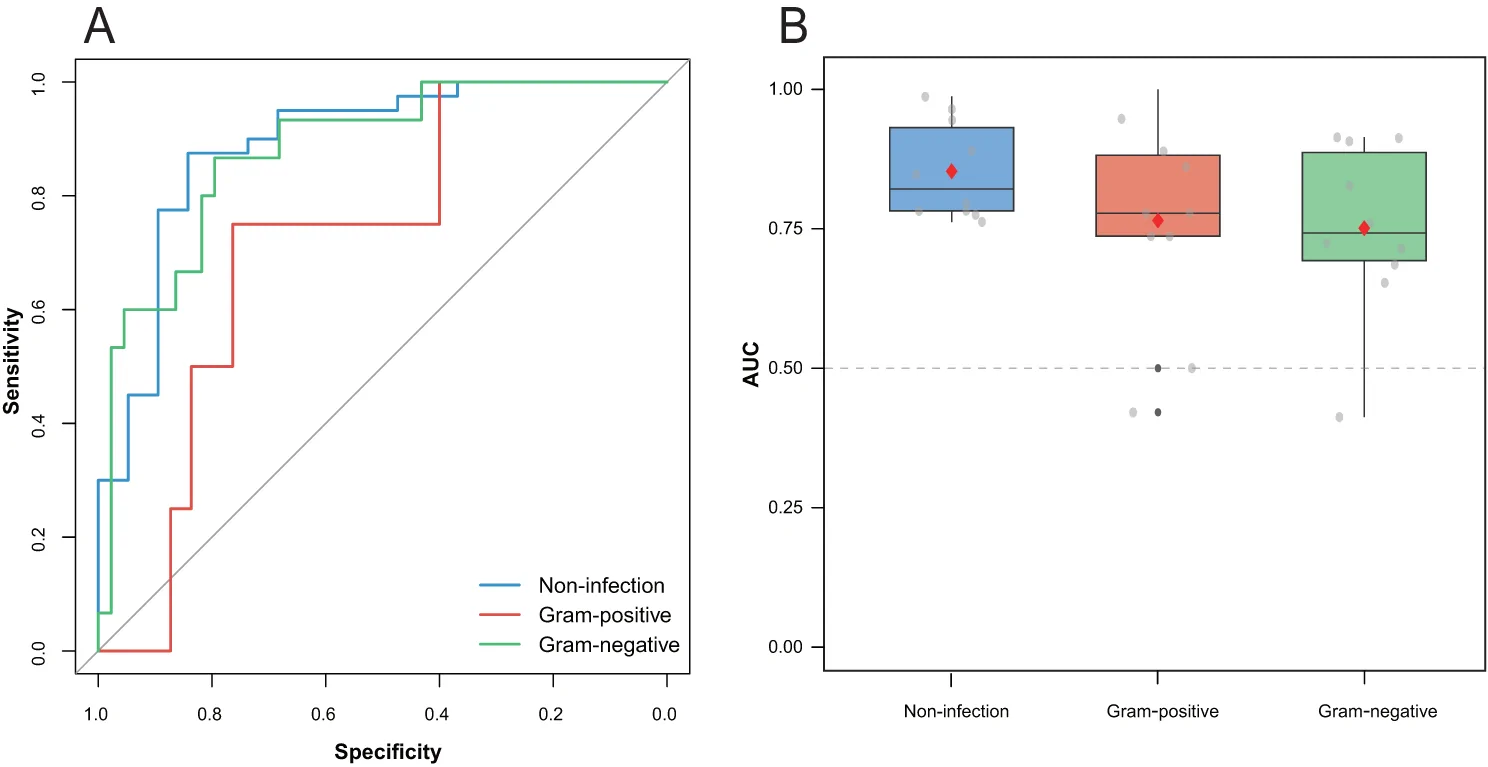
Receiver operating characteristic (ROC) curves and cross-validation results of the multinomial logistic regression model. (**A**) ROC curves for the test set: blue, red, and green curves represent non-infection, Gram-positive, and Gram-negative, respectively; (**B**) Ten-fold cross-validation area under curve (AUC) boxplot: boxplots show AUC distributions for each group; red diamonds indicate means, and gray dots represent individual fold AUC values

In the exploratory construction of a pathogen classification model, we developed a multinomial logistic regression model that encompasses three categories (non-infection, Gram-positive infection, and Gram-negative infection. However, given the limited sample size, these results should be interpreted with caution.Evaluation of the test set results revealed good discriminatory performance for the non-infected group, with an AUC of 0.89 (95% CI: 0.79–0.99) and a mean AUC of 0.85 from 10-fold cross-validation. The Gram-negative infection group achieved a test set AUC of 0.88 (95% CI: 0.78–0.98); however, its cross-validation mean AUC decreased to 0.75, indicating suboptimal stability. In contrast, the Gram-positive infection group exhibited a test set AUC of only 0.72 (95% CI: 0.49–0.94, with a notably wide confidence interval), and the cross-validation mean AUC was 0.77, accompanied by a high standard deviation of 0.18, reflecting unstable predictive performance (Fig. 1). Given the small sample size and the inherent instability of these exploratory models, we do not recommend their use for clinical decision-making without further external validation.

ROC curve analysis revealed that HBP had an AUC of 0.85 (95% CI: 0.76–0.94) for predicting severe infection, indicating discriminatory ability. Univariate logistic regression showed that each 1 ng/ml increase in HBP resulted in a 4.7% increase in the risk of severe infection (OR = 1.05, 95% CI: 1.02–1.08, *p* < 0.001). Considering the limited number of severe infection cases, this multivariable analysis was underpowered and should be interpreted with caution; the apparent reversal of the OR may reflect over-adjustment or instability due to small sample size rather than a genuine biological effect (Supplementary Figure 2).

## Discussion

This study systematically evaluated the diagnostic value of HBP, PCT, and four immune inflammatory indices (PIV, SII, SIICI, SIRI) relation to bacterial infections and PCT-defined severity stratification. We found HBP emerged as the most effective marker for distinguishing between infected and non-infection patients; its levels exhibited a progressive corresponding to the severity of infection.

HBP exhibited the most significant elevation in the infection group (53.27 vs. 19.45 ng/ml) and demonstrated superior diagnostic performance compared to PCT and all composite indices. This finding is consistent with a previous meta-analysis study that reported high pooled sensitivity and specificity for plasma HBP in diagnosing bacterial infections [30]. The optimal cut-off value of HBP in our study (approximately 40.50 ng/ml) was higher than the 15–30 ng/ml proposed by Linder et al. in emergency department sepsis patients and the 35.40 ng/ml reported by Xiao et al. in community-acquired pneumonia [31, 32]. This discrepancy is likely due to the higher proportion of severe infections (44.6%) and Gram-negative isolates (50/65) in our cohort, as Gram-negative bacteria are known to induce stronger HBP release [33]. The kinetic profiles of HBP and PCT suggest complementary clinical roles: HBP is stored in neutrophil granules and can be released within minutes to hours after bacterial stimulation, making it suitable for early screening. In contrast, PCT requires de novo synthesis, peaks at 6–24 hours, and correlates with bacterial burden, making it better suited for monitoring treatment response and guiding antibiotic de-escalation [34, 35]. However, a single time-point measurement of HBP may underestimate its clinical value. A previous prospective study demonstrated that the 48-hour change in HBP predicted 30-day mortality with an AUC of 0.82, significantly outperforming baseline HBP (0.79) and PCT (0.72) [16, 36]. Therefore, serial HBP monitoring, rather than reliance on a single measurement, should be advocated in clinical practice.

Regarding pathogen discrimination, HBP levels were significantly higher in Gram-negative infections (63.03 ng/ml) compared to Gram-positive infections (38.95 ng/ml). This observation aligns with the stronger activation of neutrophils via the LPS-TLR4 pathway induced by Gram-negative bacteria, in contrast to the TLR2 pathway activated by Gram-positive cell wall components [12]. Given that only 15 patients in our study had Gram-positive infections, this finding should be necessitate validation in larger prospective cohorts. In the context of composite immune inflammatory indices (PIV, SII, SIICI, SIRI), all indices were significantly elevated in the infection group, and SII also showing higher in the severe infection group, consistent with previous findings [21, 37, 38]. As algebraic combinations of the same base cell counts, they fail to provide independent information beyond their individual components in conventional regression models. Future strategies to address this issue may include principal component analysis, the use of simplified weighted scores (e.g., directly using the neutrophil-to-lymphocyte ratio, NLR), or incorporating composite indices as input features in machine learning models rather than treating them independent covariates in regression [22, 39, 40].

Several limitations of this study warrant acknowledgment. First, the small sample size limits the conclusiveness of our findings, making them preliminary and hypothesis-generating for empirical antibiotic selection. Second, our cohort was restricted to bacteremic patients, which constrains the generalizability of our results to non‑bacteremic infections, such as community‑acquired pneumonia, where bacteremia occurs in fewer than 10% of cases. Third, the comparison Gram-positive and Gram-negative infections was limited by a small sample size (*n* = 15 for Gram-positive). These findings are preliminary and hypothesis-generating and not robust enough to guide empirical antibiotic selection. Fourth, Validation in larger, adequately powered prospective cohorts is warranted. the retrospective design and limitations of our electronic medical record system hindered the retrieval of key clinical data, including infection source, microbiological isolates, antibiotic exposure, time from symptom onset to sampling, comorbidities, ICU admission, sepsis/septic shock status, SOFA/qSOFA scores, mortality, length of stay, and control group diagnoses. It should be noted that the definition of severe infection in this study was primarily based on laboratory parameters. The absence of comprehensive clinical parameters, such as vital signs or vasopressor requirements, may limit the comprehensiveness of this definition. This should be taken into consideration when interpreting our findings. Additionally, only a single admission blood sample was collected, precluding the analysis of dynamic changes in HBP levels. Furthermore, data on antibiotic treatment and its timing were lacking, making it impossible to assess the impact of prior therapy on marker levels. None of our prediction models were externally validated. These findings should be interpreted with caution, and future prospective, large-scale, multicenter studies that include bacteremia and non-bacteremia infections are necessary to validate our results.

## Conclusion

HBP outperforms traditional inflammatory markers in distinguishing bacterial infections from non-infectious conditions. The predictive value of HBP and PCT in distinguishing Gram‑positive from Gram‑negative infections are noteworthy. While the present single-center retrospective cohort study supports HBP as a promising diagnostic biomarker for bacterial infection, the findings regarding severity and Gram classification remain exploratory and necessitate external validation in larger, prospectively designed cohorts.

## Electronic supplementary material

Below is the link to the electronic supplementary material.

Supplementary material 1

Supplementary material 2

Supplementary material 3

Supplementary material 4

## Data Availability

No datasets were generated or analysed during the current study.

## Abbreviations

HBP: Heparin-binding protein
PCT: Procalcitonin
CRP: C-reactive protein
PIV: Pan-immune-inflammation value
SII: Systemic immune-inflammation index
SIICI: Systemic immune-inflammatory complex index
SIRI: Systemic inflammation response index
LASSO: Least absolute shrinkage and selection operator
ROC: Receiver operating characteristic
AUC: Area under the curve
CI: Confidence interval
AIC: Akaike Information Criterion
XGBoost: Extreme gradient boosting
OR: Odds ratio
ANOVA: Analysis of variance
IQR: Interquartile range
VIF: Variance inflation factor
NLR: Neutrophil-to-lymphocyte ratio
TLR4: Toll-like receptor 4
TLR2: Toll-like receptor 2
LPS: Lipopolysaccharide
