# Supplementary material for: Diagnostic utility of plasma heparin-binding protein and procalcitonin among patients with bacterial infections: a single-center retrospective study
Source: BMC Infect Dis. 2026 Aug 7;26:1949. doi: 10.1186/s12879-026-14089-6 (PMC13737857; doi:10.1186/s12879-026-14089-6)
Supplement: Supplementary file 1 [file 12879_2026_14089_MOESM3_ESM.pdf]

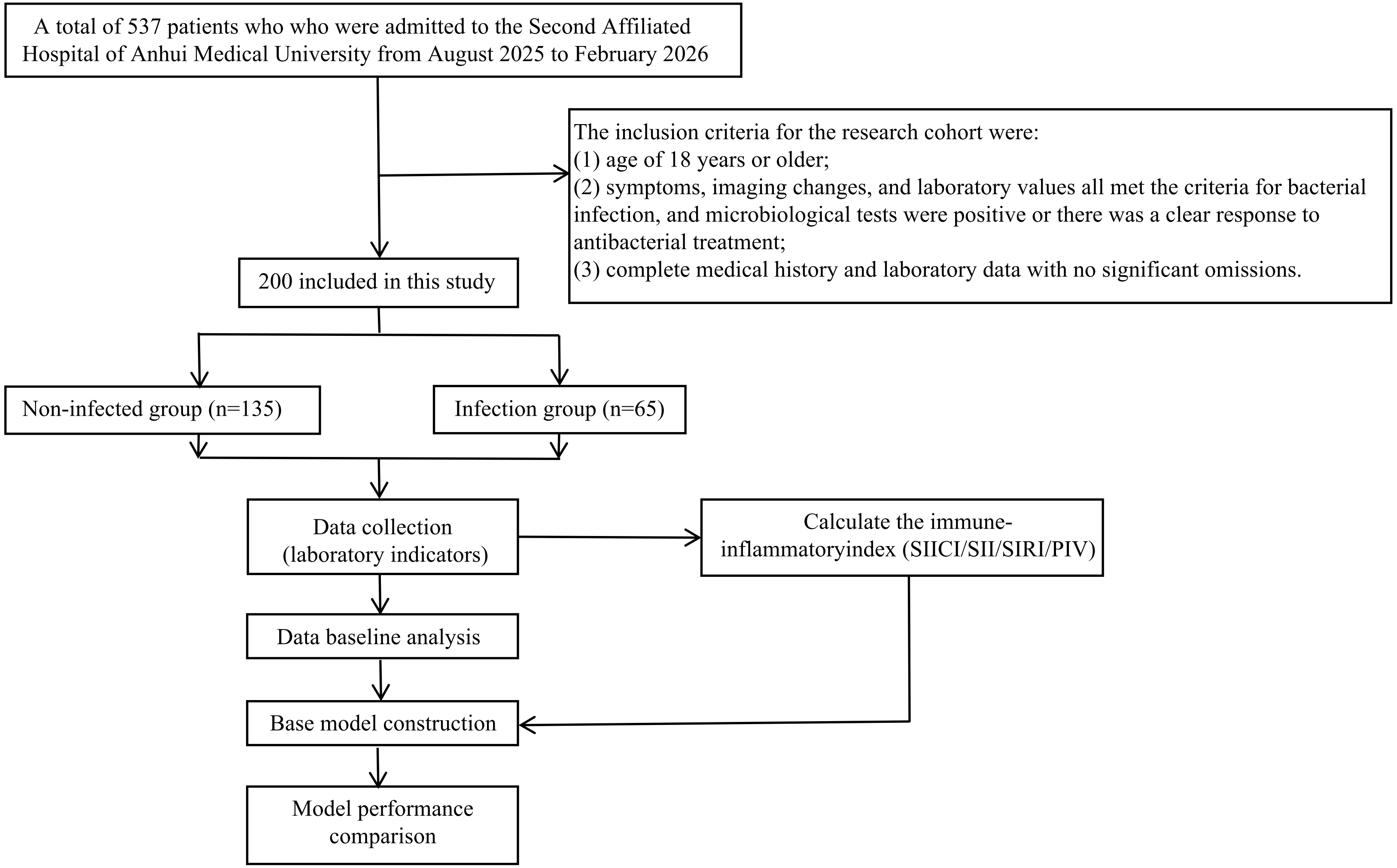
A total of 537 patients who who were admitted to the Second Affiliated Hospital of Anhui Medical University from August 2025 to February 2026
The inclusion criteria for the research cohort were:
(1) age of 18 years or older;
(2) symptoms, imaging changes, and laboratory values all met the criteria for bacterial infection, and microbiological tests were positive or there was a clear response to antibacterial treatment;
(3) complete medical history and laboratory data with no significant omissions.
200 included in this study
Non-infected group (n=135)
Infection group (n=65)
Data collection (laboratory indicators)
Calculate the immune-inflammatoryindex (SIICI/SII/SIRI/PIV)
Data baseline analysis
Base model construction
Model performance comparison
