# Supplementary material for: Diagnostic utility of plasma heparin-binding protein and procalcitonin among patients with bacterial infections: a single-center retrospective study
Source: BMC Infect Dis. 2026 Aug 7;26:1949. doi: 10.1186/s12879-026-14089-6 (PMC13737857; doi:10.1186/s12879-026-14089-6)
Supplement: Supplementary file 2 [file 12879_2026_14089_MOESM4_ESM.pdf]

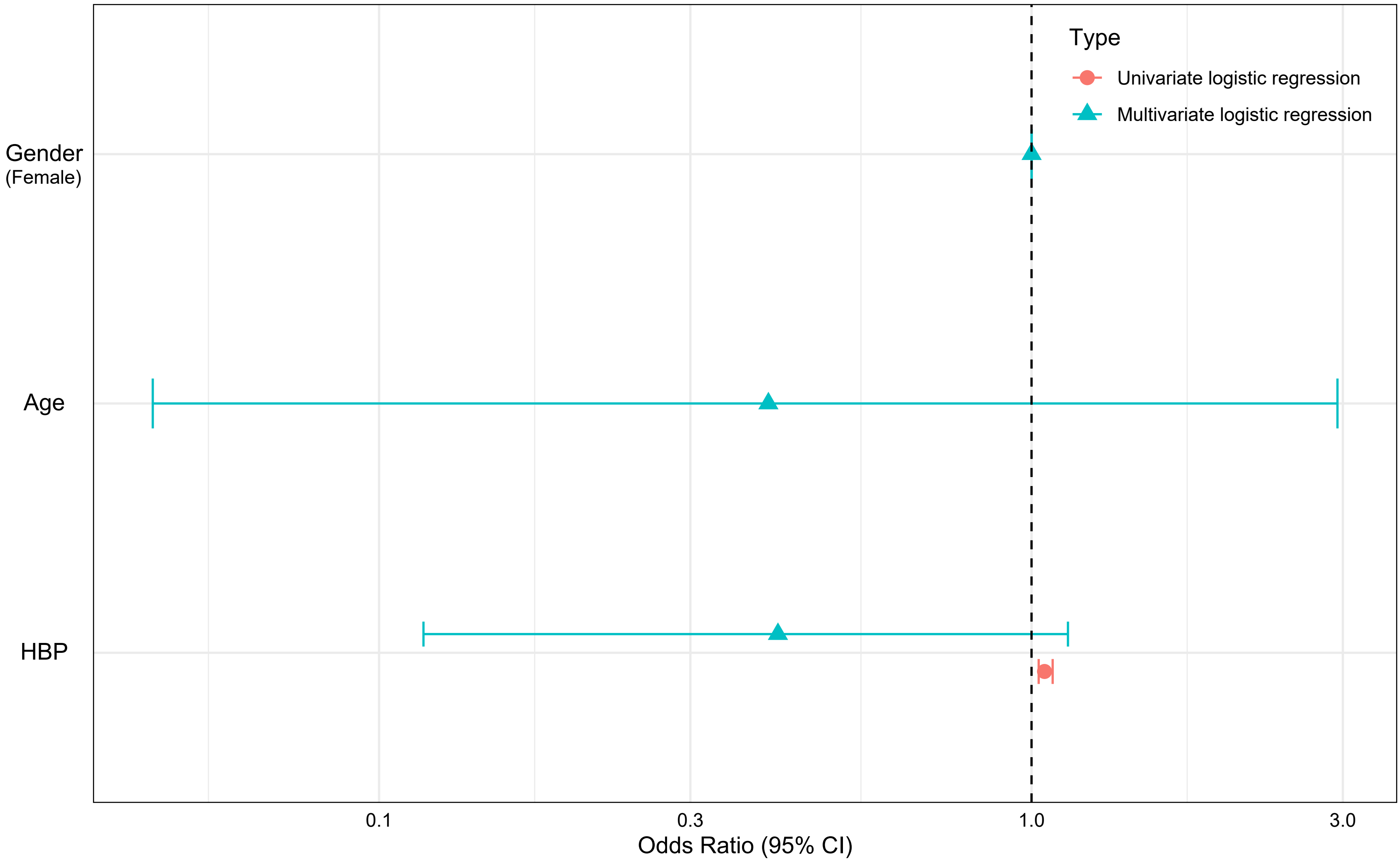

Type
Univariate logistic regression
Multivariate logistic regression
Gender
(Female)
Age
HBP
0.1
0.3
1.0
3.0
Odds Ratio (95% CI)
