# Supplementary material for: Diagnostic utility of plasma heparin-binding protein and procalcitonin among patients with bacterial infections: a single-center retrospective study
Source: BMC Infect Dis. 2026 Aug 7;26:1949. doi: 10.1186/s12879-026-14089-6 (PMC13737857; doi:10.1186/s12879-026-14089-6)
Supplement: Supplementary file 3 [file 12879_2026_14089_MOESM1_ESM.docx]

Supplementary Table 1: Comparison of clinical characteristics between patients with Gram-positive infection and Gram-negative infection.

| **Characteristics** | | | **Gram positive (*n*=15)** | | **Gram negative (*n*=50)** | |
| --- | --- | --- | --- | --- | --- | --- |
| Age (year, *x̅±s*) | | | 55.73 ± 16.81 | | 60.26 ± 13.73 | |
| Gender (*n*) | Female | 4 | | 22 | |  |
|  | Male | 11 | | 28 | |  |
| HBP (ng/ml, median, IQR) | | | 38.95 (24.88, 80.61) | | 63.03 (27.34, 114.32) | |
| PCT (ng/ml) | | | 0.46 (0.14, 1.89) | | 0.19 (0.12, 0.58) | |
| Monocyte (×10⁹/L) | | | 0.67 (0.54, 0.97) | | 0.58 (0.33, 0.81) | |
| Neutrophil (×10⁹/L) | | | 7.79 (6.05, 10.54) | | 8.06 (5.79, 11.98) | |
| Platelet (×10⁹/L) | | | 192.00 (151.00, 246.00) | | 213.00 (163.00, 290.75) | |
| Lymphocyte (×10⁹/L) | | | 1.06 (0.88, 1.64) | | 1.06 (0.74, 1.45) | |
| PIV | | | 599.22 (394.16, 1740.82) | | 695.67 (422.63, 2729.15) | |
| SII | | | 1430.38 (854.65, 1973.53) | | 1662.84 (817.27, 3210.27) | |
| SIICI | | | 22.24 (8.47, 30.14) | | 17.59 (8.30, 45.76) | |
| SIRI | | | 3.70 (2.46, 5.97) | | 4.52 (1.95, 9.18) | |

PIV: Pan-immune-inflammation Value; SII: Systemic immune-inflammation index; SIICI: Systemic immune-inflammatory complex index; SIRI: Systemic inflammation response index.
