# Supplementary material for: Diagnostic utility of plasma heparin-binding protein and procalcitonin among patients with bacterial infections: a single-center retrospective study
Source: BMC Infect Dis. 2026 Aug 7;26:1949. doi: 10.1186/s12879-026-14089-6 (PMC13737857; doi:10.1186/s12879-026-14089-6)
Supplement: Supplementary file 4 [file 12879_2026_14089_MOESM2_ESM.docx]

Supplementary Table 2: Comparison of clinical characteristics between patients with PCT-defined localized infection and severe infection.

| **Characteristics** | | **Localized infection (*n*=36)** | **Severe infection (*n*=29)** | **P** |
| --- | --- | --- | --- | --- |
| Age (year, *x̅±s*) | | 57.42 ± 14.98 | 61.45 ± 13.78 | 0.359 |
| Gender (*n*) | Female | 22 | 17 | 0.157 |
|  | Male | 14 | 12 |  |
| HBP (ng/ml, median, IQR) | | 35.13 (21.83, 56.34) | 111.95 (52.25, 133.79) | <0.001 |
| Monocyte (×10⁹/L) | | 0.66 (0.44, 0.96) | 0.58 (0.26, 0.81) | 0.076 |
| Neutrophil (×10⁹/L) | | 7.58 (5.21, 11.19) | 8.79 (6.24, 11.72) | 0.363 |
| Platelet (×10⁹/L) | | 226.50 (165.25, 266.50) | 178.00 (158.00, 287.00) | 0.356 |
| Lymphocyte (×10⁹/L) | | 1.34 (1.05, 2.04) | 0.75 (0.48, 1.05) | <0.001 |
| PIV | | 574.79 (263.92, 2341.36) | 1008.50 (499.49, 2744.12) | 0.448 |
| SII | | 968.04 (581.00, 2008.83) | 2010.49 (1529.50, 3493.95) | 0.002 |
| SIICI | | 15.94 (7.50, 27.71) | 28.08 (14.69, 58.82) | 0.062 |
| SIRI | | 2.86 (1.67, 6.23) | 5.53 (3.42, 9.94) | 0.078 |

PIV: Pan-immune-inflammation Value; SII: Systemic immune-inflammation index; SIICI: Systemic immune-inflammatory complex index; SIRI: Systemic inflammation response index.
